# “It takes more than a fellowship program”: reflections on capacity strengthening for health systems research in sub-Saharan Africa

**DOI:** 10.1186/s12913-017-2638-9

**Published:** 2017-12-04

**Authors:** Chimaraoke O. Izugbara, Caroline W. Kabiru, Djesika Amendah, Zacharie Tsala Dimbuene, Hermann Pythagore Pierre Donfouet, Esso-Hanam Atake, Marie-Gloriose Ingabire, Stephen Maluka, Joyce N. Mumah, Matilu Mwau, Mollyne Ndinya, Kenneth Ngure, Estelle M. Sidze, Charles Sossa, Abdramane Soura, Alex C. Ezeh

**Affiliations:** 1 African Population and Health Research Center, Nairobi, Kenya; 2 School of Public Health, The University of Witwatersrand, Johannesburg, South Africa; 3 Department of Demography, Faculty of Economics and Management, University of Kinshasa, Kinshasa, Democratic Republic of the Congo; 4 Department of Economic Sciences, University of Lomé, Lomé, Togo; 5 International Development Research Centre, Ottawa, Canada; 6 Institute of Development Studies, University of Dar es Salaam, Dar es Salaam, Tanzania; 7 Department of Medical Microbiology, Jomo Kenyatta University of Agriculture and Technology, Nairobi, Kenya; 8 Department of Community Health, Jomo Kenyatta University of Agriculture and Technology, Nairobi, Kenya; 9 Regional Institute of Public Health, University of Abomey-Calavi, Cotonou, Benin; 10 Ouagadougou Health and Demographic Surveillance System, ISSP, Université de Ouagadougou, Ouagadougou, Burkina Faso

## Abstract

Sub-Saharan Africa (SSA) experiences an acute dearth of well-trained and skilled researchers. This dearth constrains the region’s capacity to identify and address the root causes of its poor social, health, development, and other outcomes. Building sustainable research capacity in SSA requires, among other things, locally led and run initiatives that draw on existing regional capacities as well as mutually beneficial global collaborations. This paper describes a regional research capacity strengthening initiative—the African Doctoral Dissertation Research Fellowship (ADDRF) program. This Africa-based and African-led initiative has emerged as a practical and tested platform for producing and nurturing research leaders, strengthening university-wide systems for quality research training and productivity, and building a critical mass of highly-trained African scholars and researchers. The program deploys different interventions to ensure the success of fellows. These interventions include research methods and scientific writing workshops, research and reentry support grants, post-doctoral research support and placements, as well as grants for networking and scholarly conferences attendance. Across the region, ADDRF graduates are emerging as research leaders, showing signs of becoming the next generation of world-class researchers, and supporting the transformations of their home-institutions. While the contributions of the ADDRF program to research capacity strengthening in the region are significant, the sustainability of the initiative and other research and training fellowship programs on the continent requires significant investments from local sources and, especially, governments and the private sector in Africa. The ADDRF experience demonstrates that research capacity building in Africa is possible through innovative, multifaceted interventions that support graduate students to develop different critical capacities and transferable skills and build, expand, and maintain networks that can sustain them as scholars and researchers.

## Introduction

High-quality research capacity is a prerequisite for societal progress [1, 2]. Societies without strong research capacity face difficulties in: producing and utilizing reliable evidence for development planning and action; generating new ideas to drive economic growth and social wellbeing; deciding on trade-offs in the choice of interventions; and gauging the impact and performance of policies, programs and investments. Robust research capacity advances the production and utilization of sound evidence for decision-making and practice in all spheres of life [3]. In one of the most recent and trenchant affirmations of the importance of research capacity, the Sustainable Development Goals (SDGs)—the blueprint for global progress launched in 2015—called for concerted efforts to strengthen research and policy implementation capacities in low and middle income countries. The SDGs particularly recognize research capacity as key to: the generation of new and locally relevant knowledge; the full and safe operationalization and transfer of existing technologies; the promotion and acceleration of human, institutional and infrastructural development; and the alignment of social and health systems to deliver efficient, accessible, and affordable services to prevent and control diseases and suffering, and reduce threats, in conformity with human rights [4, 5].

### Research capacity: the sub-Saharan African situation

Sub-Saharan Africa (SSA) experiences one of the world’s severest shortages in well-trained and skilled researchers [6]. These shortages contribute to the region’s low local capacity to identify and address the root causes of its poor social, health, and other outcomes; design and drive policy innovations; and stimulate regional and national development agendas to improve development outcomes. Other consequences of weak research capacity in SSA include the low numbers of research conceptualized, conducted, and published by researchers in the region; the dearth of locally generated evidence-based ideas to tackle its myriad socioeconomic and health problems; and the region’s limited contributions to global knowledge, including high-quality patents [7–9]. Political and social support for research for health can be stimulated by developing a recognized structure for multi-partner dialogue that increases access to evidence and participation amongst stakeholders. Countries could hold annual fora where stakeholders can discuss, among other things, ways to increase evidence-based policy formulation.

Going forward, there is need to implement a communication strategy that supports the gathering, synthesis, and translation of research evidence and generates a cyclical dialogue, responsive to the diverse information needs of the different stakeholders.

The potential of researchers in SSA is curbed by several factors. These include chronic underinvestment in universities and research institutions; deficient research training and mentorship; isolation and weak scholarly networks; lack of access to current research and knowledge; low wages; and poor career prospects [10–13]. Efforts to address these challenges have taken different forms, including scholarship funds to support Africans to train in northern universities; postgraduate placement of African-trained scientists in research-active northern universities and institutions; initiatives to strengthen different elements of local graduate training programs; north-led research projects that nest Africa-based scholars, and schemes to enhance research management and productivity in universities. Many commentators argue that because these efforts have largely been driven from outside Africa and by people with limited understanding of the research capacity landscapes and issues in the continent, few of the initiatives have produced sustainable changes in the region’s research capacity needs [14–16]. For instance, scholarships for Africans to train in universities in the global north have not: created a critical mass of locally networked researchers in the region, addressed the lack of established career pathways for researchers, or strengthened local research support structures [7–9, 17]. Critics of oversea graduate training point to its particular role in draining the region of its best and most promising scholars and scientists [17, 18]. Research projects led by northern scholars also often turn African scientists into mere project managers or researchers whose scientific focus changes with the shifting research focus of their north-based collaborators (M. Mwau, personal communication, October 4, 2017).

Building sustainable research capacity in SSA calls for locally-led and run initiatives that draw on existing regional capacities as well as mutually beneficial global collaborations. In this introductory article, we describe one of such regional research capacity strengthening initiatives—the African Doctoral Dissertation Research Fellowship (ADDRF) program—an Africa-based and African-led scheme that targets doctoral students studying in universities across the SSA region. The articles in this supplemental issue of *BMC Health Services Research* are based on studies conducted by fellows of the program.

## The African doctoral dissertation research fellowship (ADDRF) program

The ADDRF program goal is to enhance the research skills of doctoral students in Africa by strengthening their scholarly competencies, expanding their scholarly networks, reducing their professional isolation, and facilitating them to complete their doctoral studies on time. The program, which is currently in its tenth year of implementation, has supported over 200 PhD students from across the region (including both Anglophone and Francophone Africa) whose research focuses on health systems, public and population health, as well as sexual and reproductive health and gender. The program has received over 5 million US dollars from the International Development Research Centre (IDRC), the Ford Foundation, the Bill & Melinda Gates Foundation, the UK Department for International Development (DFID) and Pathfinder International.

Underpinning the ADDRF program is the recognition that Africa’s development depends on her capacity to build a strong human capital base that can drive innovations and national and regional developmental efforts. However, African universities mandated to build the requisite human capital face numerous challenges that hamper their capacity [7–9]. These obstacles include the rapid growth in student enrolment and the expansion of training programs, especially at the undergraduate level, at a time when per capita funding for universities in the region is generally plummeting [7]. Many universities in the region also presently operate with overstretched and poorly-motivated faculty who resort to consultancies to maintain a basic living standard. While these problems affect university programs in Africa across the board, their impact on graduate studies, and particularly, doctoral training appears starker [7, 8, 15].

The challenges that face students in graduate programs in SSA include poor quality supervision, limited exposure to current research methods, literature, and debates as well as weak academic and research environments. Many graduate students also lack role models and mentors, strong academic and research networks, opportunities to participate in international conferences, and exposure to strong research systems. In addition, majority of graduate students on the continent lack funding for their studies and are forced to work part-time, consequently taking many years to graduate [7] or even never completing their studies.

### The smell of success

Early on, the initiators of the ADDRF recognized that different interventions were needed at multiple levels and fronts to ensure the success of program fellows. Timely throughput of quality PhDs is vital, but not always enough to convert PhD graduates into productive researchers and research leaders [14]. The ADDRF program selects its fellows competitively, which guarantees that it attracts the best and most promising students. Fellows are offered various forms of support at different stages of their PhD journey. These include research methods and scientific writing workshops, research grants, post-doctoral research support and placements as well as sponsorship to network and attend scientific conferences. ADDRF writing workshops offer fellows protected time where experienced researchers and global experts provide hands-on support and review their manuscripts to guarantee publications in high-quality journals. These workshops are also delivered using creative pedagogical methods that equip fellows with vital scholarly and transferable research skills that enable them to: do quality research; keep to PhD completion timelines; engage with researchers from across multiple but related disciplines; cultivate research networks and collaborations; and develop research leadership skills. Innovative strategies such as prizes for joint publications and small grants for collaborative research projects stimulate inter- and intra-cohort collaboration among fellows. Support for conference participation helps fellows form and extend their scholarly networks. In addition, since 2014, the program has pioneered attractive re-entry research grants and postdoctoral fellowships to launch fellows’ research careers and sustain their interest and skills in research.

The ADDRF program has made important differences in the work, capacity, and research of its fellows. Two independent successive tracer studies and evaluations of the program highlighted a number of positive impacts. These include that over 90% of the fellows who had graduated with their PhDs were residing and working in their countries of origin; that most of these graduates remain active in research or and teaching at the university level and have at least two publications in peer-reviewed journals; and that the program had expanded the fellows’ scholarly networks and created cohorts of actively networked multidisciplinary researchers. Further, successive cohorts of fellows continue to report improved technical knowledge in their fields, enhanced communication skills, and observable improvements in personal competences related to research following participation in the ADDRF program, including fundraising for research. Collectively, ADDRF fellows have published over 350 articles in peer-reviewed journals and book chapters. Majority of them have also received additional funding support for their research.

The ADDRF has emerged as a practical and tested innovation for producing African research leaders, strengthening university-wide systems for quality research training and productivity, and building a critical mass of highly-trained African academics at the PhD level. Across the region, ADDRF fellows and graduates are emerging as research leaders, showing signs of becoming the next generation of world-class researchers. The program has furnished them with new skills in: developing research networks and groups, financial management, research stewardship, grantsmanship, information technology for research, referencing, graduate supervision and mentorship. Although the large number of countries and institutions in the region limits the ability of the program to make significant direct investments in building institutions and universities, ADDRF fellows, many of whom are university faculty, are already emerging as future leaders and change agents in their institutions. These scholars and researchers are supporting the production of the next generation of researchers in their institutions and driving the transformations of these institutions into research-active hubs.

While the contributions of the ADDRF program to research capacity strengthening in the region are significant, our experience with the program highlights critical gaps that require concerted efforts to fill. First, funding for research capacity strengthening mainly comes from the global north. Yet, the sustainability of the ADDRF and other research and training fellowship programs on the continent requires significant investments from local sources and, especially governments and the private sector in Africa. Second, research capacity across the continent is uneven: competitive programs, such as the ADDRF, may inadvertently exacerbate those gaps through their selection processes, further widening the scientific knowledge and capacity inequality between different countries and sub-regions on the continent. Such differences should be considered in the design and delivery of research capacity strengthening programs. In particular, efforts must go into closing gender gaps in graduate training in Africa as well as addressing disparities in high quality research capacity among Anglophone, Francophone, and Lusophone African countries.

## Concluding thoughts

Sub-Saharan Africa faces many developmental challenges that can be addressed by strengthening local research capacity. The ADDRF story demonstrates that research capacity building in Africa is possible through innovative and well-thought out capacity-strengthening strategies. The future holds immense potential for both the ADDRF program and its fellows. Our fellows across the continent are evidence that substantive and sustainable institutional transformation at African universities must come from within, through a critical mass of researchers who are willing, ready, able, and supported to drive change. Training and retaining promising researchers within Africa is the crucial first step to achieving sustainable research capacity for the region. However and as we have shown in this paper, research capacity strengthening requires a lot more than supporting individuals to acquire PhDs. It requires sustained engagement to enable them build requisite capacities, transferable skills, and networks that can sustain them as scholars and researchers. More importantly, it invites a clear-eyed appreciation of the challenges scholars face in the region and the arming of emerging researchers with a miscellany of competences and skills to sustainably surmount these barriers. To end with the apt words of a staffer of one of the organizations that fund the ADDRF program: “it takes more than a fellowship program to strengthen research capacity and build research leaders”.

## References

[1] Hasnida A, Borst RA, Johnson AM, Rahmani NR, van Elsland SL, Kok MO (2017). Making health systems research work: time to shift funding to locally-led research in the south. Lancet Glob Health.

[2] Watson R, Crawford M, Farley S (2003). Strategic approaches to science and technology in development.

[3] Cooke J, Owen J, Wilson A (2002). Research and development at the health and social care interface in primary care: a scoping exercise in one National Health Service region. Health Soc Care Community.

[4] United Nations. Goal 17: Revitalize the global partnership for sustainable development. http://www.un.org/sustainabledevelopment/globalpartnerships/. Accessed 12 Jan 2017.

[5] United Nations General Assembly. Transforming our world: The 2030 agenda for sustainable development. 2015 https://sustainabledevelopment.un.org/content/documents/21252030%20Agenda%20for%20Sustainable%20Development%20web.pdf. Accessed 1 Oct 2017.

[6] Tettey WJ (2006). Challenges of developing and retaining the next generation of academics.

[7] Ezeh AC, Izugbara CO, Kabiru CW, Fonn S, Kahn K, Manderson L, Undieh AS, Omigbodun A, Thorogood M (2010). Building capacity for public and population health research in Africa: the consortium for advanced research training in Africa (CARTA) model. Glob Health Action.

[8] Kabiru CW, Izugbara CO, Wairimu J, Amendah D, Ezeh AC (2014). Strengthening local health research capacity in Africa: the African doctoral dissertation research fellowship program. Pan Afr Med J.

[9] Kabiru CW, Izugbara CO, Wambugu SW, Ezeh AC (2010). Capacity development for health research in Africa: experiences managing the African doctoral dissertation research fellowship program. Health Res Policy Syst.

[10] Blair R, Jordan J (1994). Retaining teaching capacity in African universities: problems and prospects.

[11] ESSENCE On Health Research (2014). Seven principles for strengthening research capacity in low-and middle-income countries: simple ideas in a complex world. ESSENCE good practice document series.

[12] Wight D (2005). Impediments to developing social science research capacity in East Africa (occasional paper 14).

[13] Wight D (2008). Most of our social scientists are not institution based… they are there for hire—research consultancies and social science capacity for health research in East Africa. Soc Sci Med.

[14] Fonn S (2006). African PhD research capacity in public health: raison d’etre and how to build it. Global Forum for Health Research.

[15] Fonn S, Egesah O, Cole D, Griffiths F, Manderson L, Kabiru C, Ezeh A, Thorogood M, Izugbara C (2016). Building the capacity to solve complex health challenges in sub-Saharan Africa: CARTA’s multidisciplinary PhD training. Can J Public Health.

[16] Sam-Agudu NA, Paintsil E, Aliyu MH, Kwara A, Ogunsola F, Afrane YA, Onoka C, Awandare GA, Amponsah G, Cornelius LJ (2016). Building sustainable local capacity for global health research in West Africa. Ann Glob Health.

[17] Arowosegbe JO (2016). African scholars, African studies and knowledge production on Africa. Africa.

[18] Sewankambo NK, Cooper AF, Kirton JJ, Lisk F, Besada H (2016). Strengthening health capacity in sub-Saharan Africa: a millennium development challenge. Africa's health challenges: sovereignty, mobility of people and healthcare governance.

